# Chitinases Are Negative Regulators of *Francisella novicida* Biofilms

**DOI:** 10.1371/journal.pone.0093119

**Published:** 2014-03-24

**Authors:** Myung-Chul Chung, Scott Dean, Ekaterina S. Marakasova, Albert O. Nwabueze, Monique L. van Hoek

**Affiliations:** School of Systems Biology and the National Center for Biodefense and Infectious Diseases, George Mason University, Manassas, Virginia, United States of America; University of Alberta, Canada

## Abstract

Biofilms, multicellular communities of bacteria, may be an environmental survival and transmission mechanism of *Francisella tularensis.* Chitinases of *F. tularensis* ssp. *novicida* (*Fn*) have been suggested to regulate biofilm formation on chitin surfaces. However, the underlying mechanisms of how chitinases may regulate biofilm formation are not fully determined. We hypothesized that *Fn* chitinase modulates bacterial surface properties resulting in the alteration of biofilm formation. We analyzed biofilm formation under diverse conditions using chitinase mutants and their counterpart parental strain. Substratum surface charges affected biofilm formation and initial attachments. Biophysical analysis of bacterial surfaces confirmed that the *chi* mutants had a net negative-charge. Lectin binding assays suggest that chitinase cleavage of its substrates could have exposed the concanavalin A-binding epitope. *Fn* biofilm was sensitive to chitinase, proteinase and DNase, suggesting that *Fn* biofilm contains exopolysaccharides, proteins and extracellular DNA. Exogenous chitinase increased the drug susceptibility of *Fn* biofilms to gentamicin while decreasing the amount of biofilm. In addition, chitinase modulated bacterial adhesion and invasion of A549 and J774A.1 cells as well as intracellular bacterial replication. Our results support a key role of the chitinase(s) in biofilm formation through modulation of the bacterial surface properties. Our findings position chitinase as a potential anti-biofilm enzyme in *Francisella* species.

## Introduction

Many bacteria including bacterial pathogens live in muticellular communities, called biofilms, on abiotic and biotic surfaces [1]–[3]. Biofilms have characteristic architectural and phenotypic properties including the creation of sticky extracellular matrix, consisting of proteins, lipids, extracellular DNA (eDNA), and exopolysaccharides (EPS) to mediate surface attachment, intercellular adhesion, biocide resistance, and immune evasion [4]. Biofilm matrix alters bacterial sensitivity to chemical attack [5], causing phenotypic antibiotic resistance.

*Francisella tularensis* is a Gram negative, facultative intracellular pathogen that causes tularaemia. It is considered a category A agent by the Centers for Disease Control and Prevention (CDC) due to its high infectivity, dissemination by aerosol and high mortality to humans. In environmental conditions, *F. tularensis* Type B (*holarctic*) is associated with water and waterways and infects many species of animals, insects, and protists. Our previous study showed that *F. tularensis* ssp. *novicida* (*Fn*), a model strain for the more virulent *F. tularensis* [6], is able to form an *in vitro* biofilm [7]. A recent study demonstrated that *Fn* forms biofilms on chitin surfaces, and this activity is dependent on chitinases, the Sec secretion system, and several Sec-dependent secreted proteins, some of which are predicted to bind and/or degrade chitin [8]. Since *Fn* is associated with water-borne transmission, biofilm formation is likely linked to its environmental persistence in aquatic habitats [7], [9], [10], as well as possibly within tick and mosquito vectors that have chitin in their exoskeletons [8], [11]. However, the role of chitinases in *Francisella* biofilm formation is not known.

Chitinases are glycosyl hydrolases that hydrolyze chitin, a linear β-1,4-linked polymer of N-acetyl-D-glucosamine (GlcNAc), the second most abundant polysaccharide in nature after cellulose. Chitinases are found in a wide range of species [12]–[14], including those that are known not to synthesize chitin, such as bacteria, viruses, higher plants as well as mammals. Based on the cleavage site on chitin of the chitinolytic enzymes, chitinases are divided into exo-chitinases and endo-chitinases [15]. Endo-chitinases cleave chitin randomly at internal sites, generating soluble oligomers (2∼4 units of GlcNAc). Exo-chitinases such as chitobiosidases and β-(1,4)-N-acetyl-glucosaminidases act on the non-reducing end of chitin to digest into (GlcNAc)_2_ and GlcNAc, respectively [15]. In *Francisella,* four putative chitinases (ChiA, ChiB, ChiC, and ChiD) were identified and characterized *in vitro* using biochemical studies coupled with bioinformatics analyses [16]. Enzymatic analyses revealed that chitinases ChiA and ChiB possessed both endo- and exo-chitinase activity. *Fn* thus has two functional chitinases ChiA and ChiB, despite having all four chitinase genes in the genome [16]. Although biofilm formation of *Fn* on chitin was shown to be dependent on the two chitinase genes, *chiA* and *chiB* [8], the underlying mechanisms of how chitinases regulate biofilms are not fully determined.

In this study, we hypothesized that *Fn* chitinase changes the contents and/or composition of its EPS, resulting in altered biofilm formation. Studies using transposon-inserted *chi* mutants and exogenous chitinase showed that chitinase is a negative regulator of *Francisella* biofilm formation and causes dispersion of pre-formed biofilms, and alters bacterial surface properties. Our results provide a basis for understanding the mechanism of biofilm dispersion that may be applicable to a large number of biofilm-forming pathogenic species. Insights into the mechanism of chitinase function have implications for the control of biofilm-related infections.

## Results

### Effect of chitinases on biophysical properties of the bacterial surface

**Figure 1:**
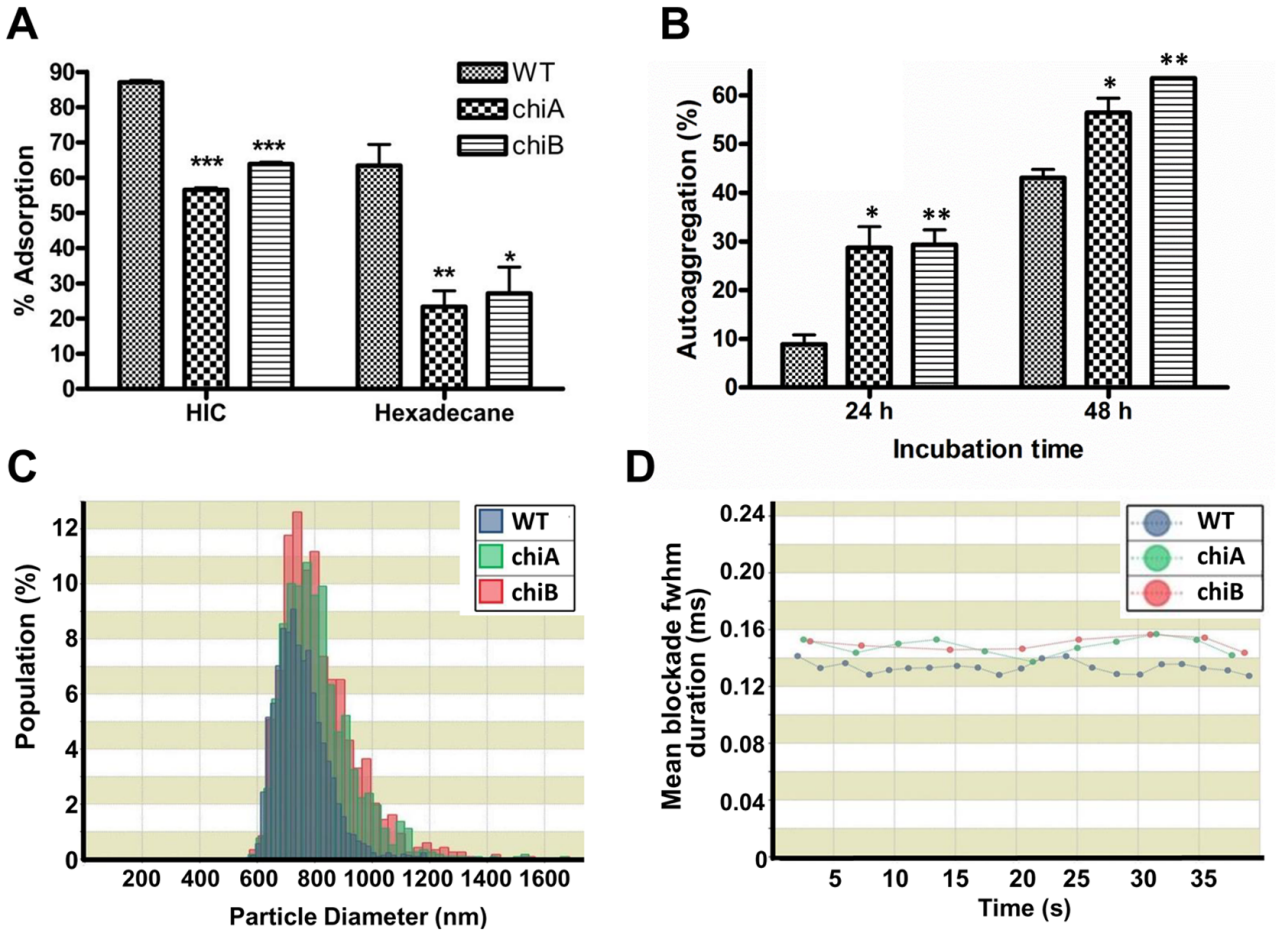
*Fn* chitinase affects the biophysical properties of the biofilm. *Fn* was grown to mid-log phase prior to the analyses. (**A**) The relative hydrophobicity of WT and *chi* mutants assayed by phenyl-sepharose column chromatography (HIC) and microbial adhesion to the nonpolar solvent hexadecane. *P<0.05, **P<0.01, and ***P<0.001 compared to WT (n = 6). (**B**) Autoaggregation of WT and *chi* mutants in PBS assayed at 24 and 48 h. *P<0.05, and **P<0.01 compared to WT (n = 6). (**C**) Size distribution for planktonic cultures of the strains in PBS measured by qNano analysis. (**D**) Particle translocation time (fwhm). The *chi* mutants had a larger fwhm duration than that observed for WT, indicating that the lower charge *chi* mutants took longer to traverse the pore. Mean presented in dots was calculated from every 100 data points.

To examine a role of *Fn* chitnases on biofilm formation, we analyzed the biophysical properties of the bacterial surfaces of WT and transposon insertion mutants in *chiA* and *chiB* gene. In the Hydrophobic Interaction Chromatography (HIC) and Microbial Adhesion To Hydrocarbon (MATH) analysis [17], [18], the *chi* mutants had a lower adsorption activity to the phenyl-sepharose and to the nonpolar hydrocarbon hexadecan than WT, respectively (Fig. 1A). The *chi* mutants always precipitated faster than WT cells in the autoaggregation study. After 48 h, the autoaggregation of the *chi* mutants reached >60%, while that of WT was ∼43% (Fig. 1B). The size tunable pore sensor qNano utilizes a non-optical detection principle to determine the size, concentration, dynamics and charge of a wide range of particle types [19]–[21]. To analyze bacterial sizes and surface charges, we used the qNano nanoparticle characterization system with planktonic bacteria cultured overnight. Increased bacterial sizes were observed in the *chi* mutants (Fig. 1C). In addition, the *chi* mutants showed a longer pore translocation times than WT (Fig. 1D), suggesting that *chi* mutants are less net negative-charged during planktonic growth. These results suggest that mutation of *chi* genes changes biophysical properties of *Fn* [22], [23].

For complementation *in trans*, we attempted to clone the *chiA* and *chiB* genes into plasmid pKK214 containing the *groEL* promoter of the *F. tularensis* live vaccine strain. Unfortunately, we could not obtain pKK214-*chiA* for complementation, probably due to technical difficulties such as a larger size of the insert.

### Effect of substratum surface charges on biofilm formation and initial attachment

**Figure 2:**
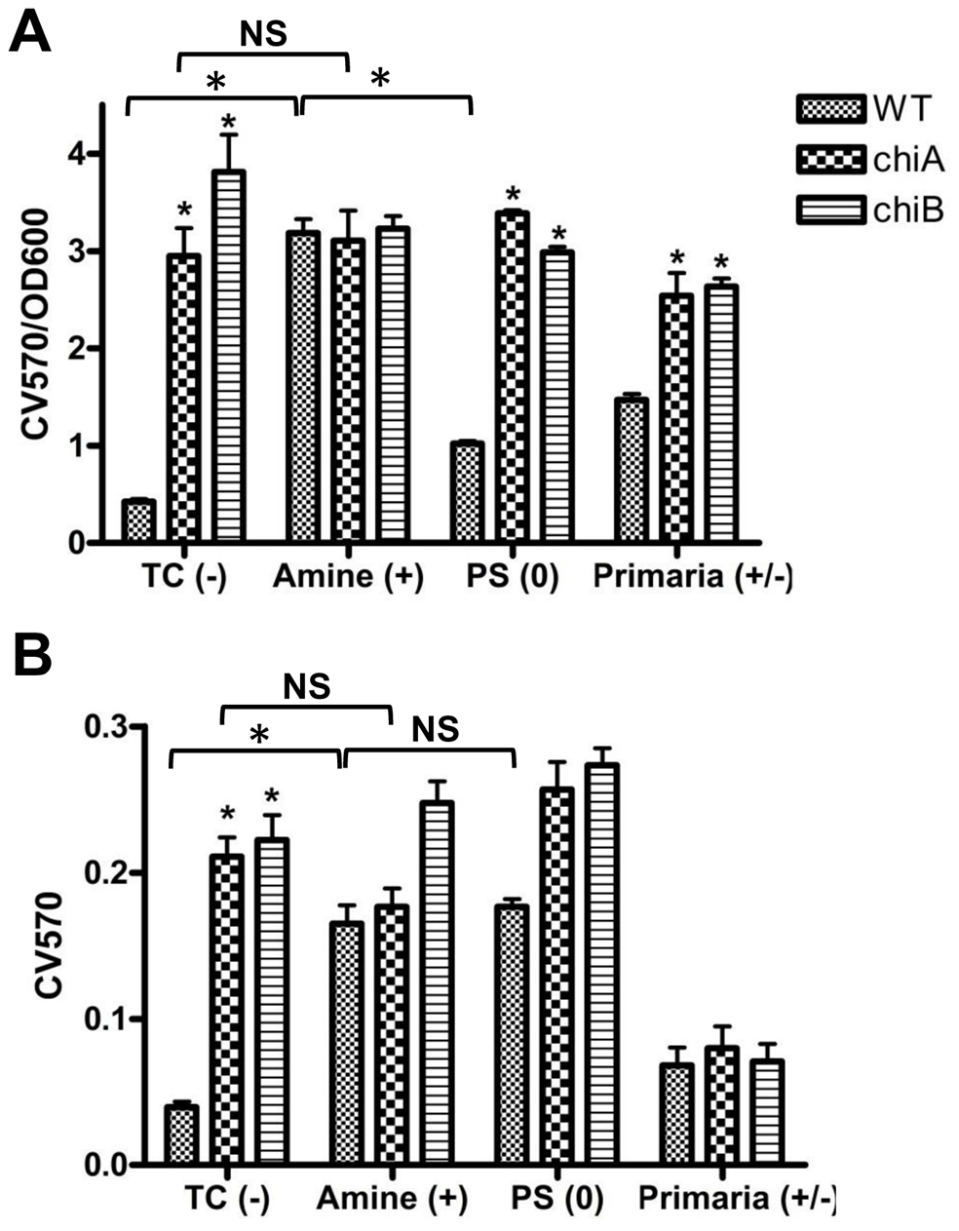
*Fn* chitinase affects biofilm formation in different surface charged microplates. (**A**) Biofilm formation based on CV staining (CV570) of cells adherent to negatively (TC), positively (Amine), neutral (PS) and positively/negatively (Primaria) charged 96-well plates, normalized by bacterial growth (OD600) expressed as CV570/OD600. (**B**) Attachment was assessed by CV staining 1 h post-inoculation of stationary-phase cultures (OD = 1.0). Initial attachment of *Fn* WT was very low to the TC and Primaria plates, but high to the amine and PS. *P<0.01 (n = 6) and NS (not significant) by unpaired Student's t-test.

We examined the effect of substratum surface properties on *Fn* biofilm formation of wild type *Fn* (WT) and *chi* mutants to substrata with different surface charge properties. Different types of microplates including tissue-culture treated (TC), non-treated (PS), amine treated and Primaria surface-modified polystyrene plates were used for negative, hydrophobic neutral, positive and positive/negative-charges, respectively. Biofilm formation of WT on a positively-charged amine microplate was significantly higher than on a negatively-charged tissue-culture plate with P<0.01 (Fig. 2A). There were no significant differences of bacterial growth in different types of microplates (data not shown). However, biofilm formation of WT on non-treated PS and Primaria plate was comparable to that on TC plate, suggesting that only a positive-charged substratum surface affected WT *Fn* biofilm formation

The first step of biofilm formation is adhesion to a surface. This is mediated by many factors including the charge of the substratum surface and the charge of the bacteria. To determine the potential effect of surface charges on bacterial attachment, we determined the capacity of WT and *chi* mutants for attachment to different surface charged plates using a 1-h attachment assay. Initial attachment of *Fn* WT was very low to the TC (−) and Primaria (+/−) plates, but high to the amine (+) and PS (0), indicated by CV staining (Fig. 2B). The *chi* mutants showed higher initial attachment to the TC, amine, and PS, but not to the Primaria plate. Relative initial attachment of *chi* mutants was higher than that of WT in a negatively-charged TC plate (Fig. S1), suggesting that in wild-type *Fn*, chitinases are involved in increasing charge of the bacterial surface, and promoting attachment to negatively-charged surfaces (Fig. 1D). For chitinase mutants, attachment appears to be independent of surface charge. Therefore, we hypothesize that altered production of EPS may also be contributing to the differences in *chi* mutant biofilm production through increase in hydrophobicity.

### COMSTAT2 analysis of biofilms

**Figure 3:**
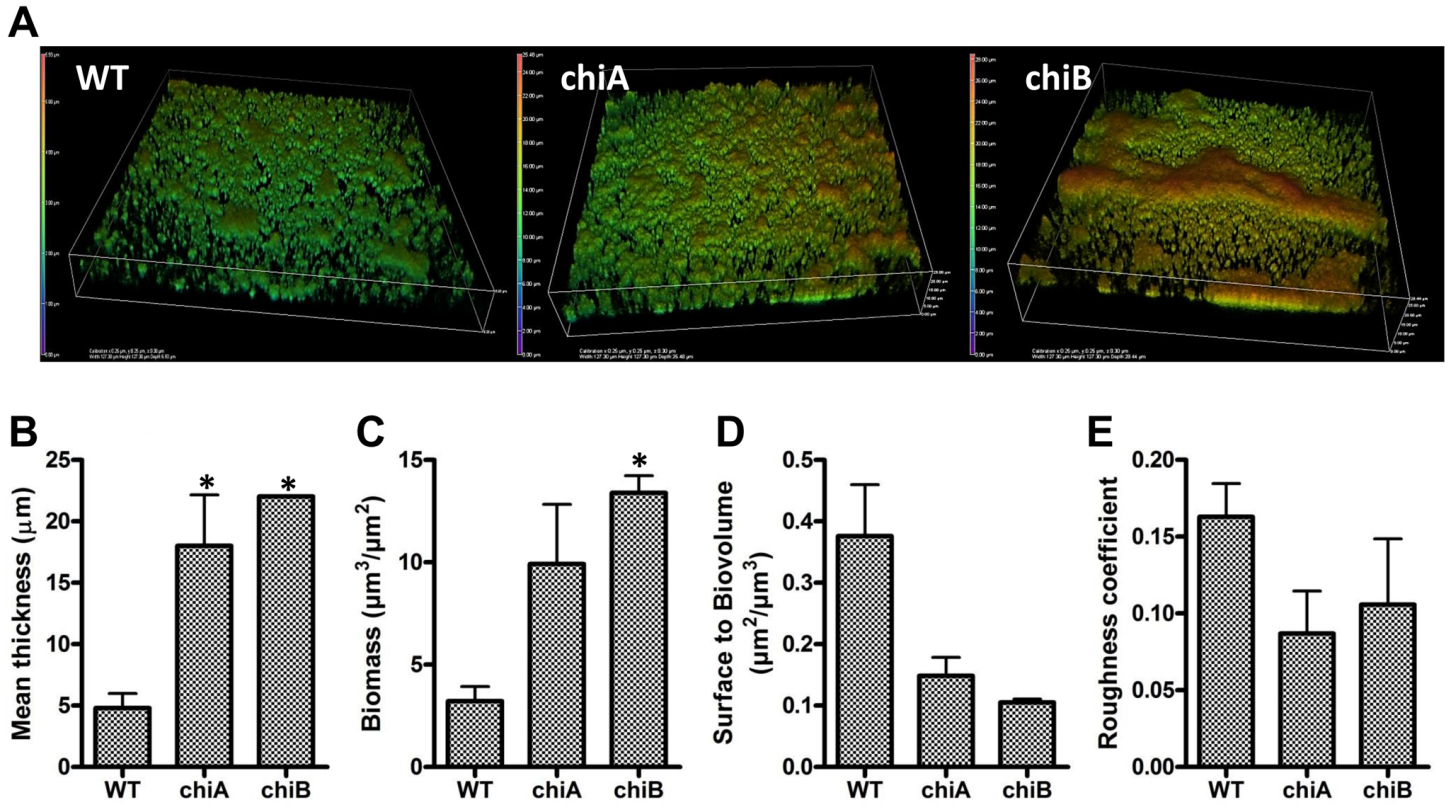
COMSTAT2 analysis of WT and *chi* mutants. Biofilms were grown in LabTek II glass chambers for 24-U confocal microscope. (**A**) 3D structures of biofilms were analyzed by CLSM z-stacks and z-stacks were rendered using Bitplane Imaris software. The images shown are representative of three independent experiments. (**B**) Mean thickness, (**C**) biomass, (**D**) surface to volume ratio, and (**E**) roughness coefficient of biofilms. *P<0.05 (n = 3) by unpaired Student's t-test.

When compared to WT biofilm formation, *chiA* and *chiB* mutants showed a significant increase in biofilm formation for both the TC and non-treated PS plate. In microscopic analysis with CV staining, WT did not show prominent 3D bacterial communities on the TC plate the surface (Fig. S1). *Chi* mutants displayed significant 3D biofilm architectures on the negative charged borosilicate glass (Fig. 3A, Fig. S1D) and in the TC plate (Fig. S1C). COMSTAT2 analysis confirmed that mutation of *chi* genes resulted in a significant increase in mean thickness and biomass of biofilms (Fig. 3B and 3C). The ratio of surface to biovolume for WT biofilms was 2.5- or 3.6-fold higher than the ratio for induced *chiA* or *chiB* biofilms, respectively (Fig. 3D). This indicated that WT *Fn* formed flat, undifferentiated biofilms that covered 2.6 or 3.5 times more surface (with the same amount of biomass) than *chi* mutant biofilms. These results suggest a *chi*-dependent regulation of *Fn* biofilm formation, such that the ability to produce chitinase leads to an overall decrease in biofilm structure and architecture (i.e. chitinase mutants produce a hyper-biofilm structure). This effect may be partly due to altered attachment, suggesting that EPS acts as an adhesin involved in cell-to-surface interactions [24], [25].

### Lectin binding assay for identification of potential chitinase substrate

**Figure 4:**
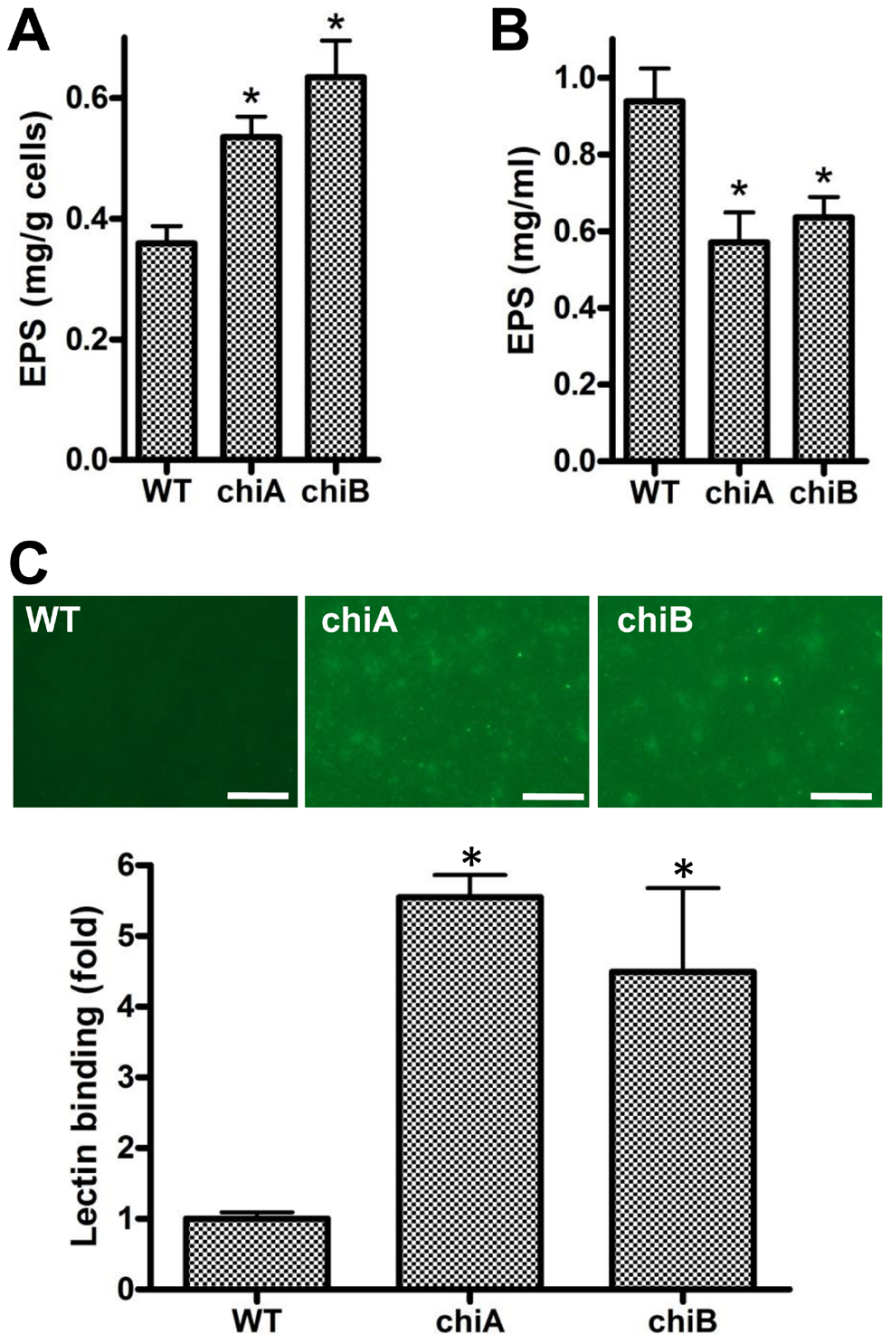
(**A**) EPS contents of the cells and (**B**) culture supernatants of the strains. EPS contents were determined by phenol extraction followed by phenol-sulfuric acid method for carbohydrates as described in Materials and Methods. (**C**) Lectin binding assay to biofilms. FITC-Con A and FITC-WGA lectins were used for biofilm binding. Lectin binding capacity to biofilms was measured by a fluorescence plate reader and calculated relative fold to WT binding. Fluorescence microscopic images of biofilms of WT, *chi*A and *chi*B grown in TC plate are shown in the top panel. Biofilms in the TC plate were shown by CV staining (Fig. S1C). Scale bar, 100 μm.

To determine if chitinase is involved in the EPS production, the EPS contents of cells and culture supernatants of the three strains were determined by a phenol-sulfuric acid method. Total EPS contents of the *chi* mutant cells were higher than that of WT cells, while total EPS content in WT culture supernatants was higher than those of the mutant culture supernatants (Fig. 4A and 4B). These results support the findings that the *chi* mutants have a thick EPS, contributing to the bigger size of the cells as observed in Fig. 1C.

The structure or composition of biofilm EPS can be partly deduced on the basis of the specific binding of lectins to different sugar residues [26]. To identify a potential chitinase substrate, binding of the FITC-labeled lectin concanavalin A (ConA) to the biofilms of three strains was fluorospectrometrically analyzed in a TC microplate. The binding of ConA to *chiA* and *chiB* mutants, but not to WT (Fig. 4), demonstrates that fluorescence from ConA is closely associated with the cells. Fluorescence microscopic analysis further supported the binding of ConA to the mutants. These results suggest that chitinase cleavage of its substrates could have exposed the ConA-binding epitope (e.g. mannose α1-3- or α1-6-containing EPS) in *Fn* biofilms.

### Requirement of chitinase activity for its anti-biofilm property

**Figure 5:**
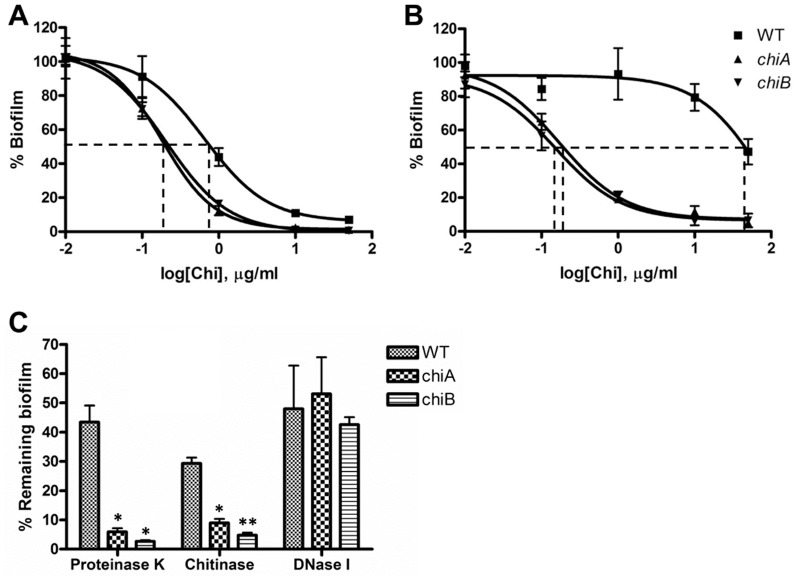
Enzymatic activity of chitinase is required for regulation of *Fn* biofilm formation. (**A**) Effect of exogenously added chitinase on biofilm formation in the negatively-charged TC plates. EC_50_s of exogenous chitinase to WT, *chiA* and *chiB* mutants were determined to be 0.65, 0.18, and 0.21 μg/ml, respectively (n = 6). (**B**) Effect of exogenous chitinase on biofilm formation in the positively-charged amine plates. EC_50_s of chitinase to WT, *chiA* and *chiB* mutants were determined to be 87.46, 0.17, and 0.15 μg/ml, respectively (n = 6). (**C**) Detachment of *Fn* biofilms after exposure to proteinase K, chitinase and DNase I (50 μg/ml) in the TC plates. Untreated control CV_570_ values were 0.149±0.032, 0.588±0.012, and 0.585±0.017 for *Fn* WT, *chiA* and *chiB* mutants, respectively. *P<0.01 and **P<0.001 compared to control without enzyme treatment (n = 6).

Since chitinase appeared to modulate biofilm formation, we tested whether chitinase activity itself is responsible for the anti-biofilm activity. To test the effect of exogenous chitinase on biofilm formation, bacteria were incubated with *Streptomyces griseus* chitinase, both because *S. griseus* chitinase is comparable to *Fn* chitinase in three chitinase activities, e.g. chitobiosidase, β-N-acetylglucosaminidase, and endochitinase activity (Fig. S2) [16], and because enzymatic activity of chitinase contributes to its antibiofilm activity as demonstrated below. *Fn* WT showed slightly higher chitinase EC_50_ (0.65 μg/ml) in the TC (−) plate compared with the *chi* mutants (0.18 and 0.21 μg/ml for *chiA* and *chiB*, respectively) (Fig. 5A) consistent with the increased biofilm formation in *chiA*. On the other hand, on amine (+) plate, EC_50_ of exogenous chitinase to WT was dramatically increased (87.46 μg/ml), while EC_50_ of exogenous chitinase to the *chi* mutants did not show significant changes (0.17 and 0.15 μg/ml for *chiA* and *chiB*, respectively) (Fig. 5B). The *chi* mutants showed high biofilm formation with no significant difference (Fig. 2A) on TC and amine plates. Exogenous chitinase also significantly affected biofilm formation of the Gram-negative pathogen *P. aeruginosa.* Exogenous chitinase could affect biofilm formation of *S. aureus*, but only if much higher concentration was used (Fig. S3).

We next examined whether pre-formed biofilms can be detached from plastic surfaces by enzymatic degradation of the matrix polymers. Biofilms were grown in the wells of 96-well TC plates and then treated with different test enzymes for 2 h at a final enzyme concentration of 50 μg/ml. Treatment with proteinase K, chitinase, and DNase I resulted in a significant decrease in remaining biofilms of all three strains assayed by CV staining (Fig. 5C), suggesting these enzymes partially degraded matrix materials. Interestingly, chitinase had by far the greatest effect on biofilm of the *chi* mutants, causing over 90% reduction in CV staining, suggesting that chitinase substrate polysaccharides are a major structural component of the biofilm. However, this finding suggests that proteins and eDNA are also important components of the *Fn* biofilms.

### Effect of chitinase inhibitors on biofilm formation

**Figure 6:**
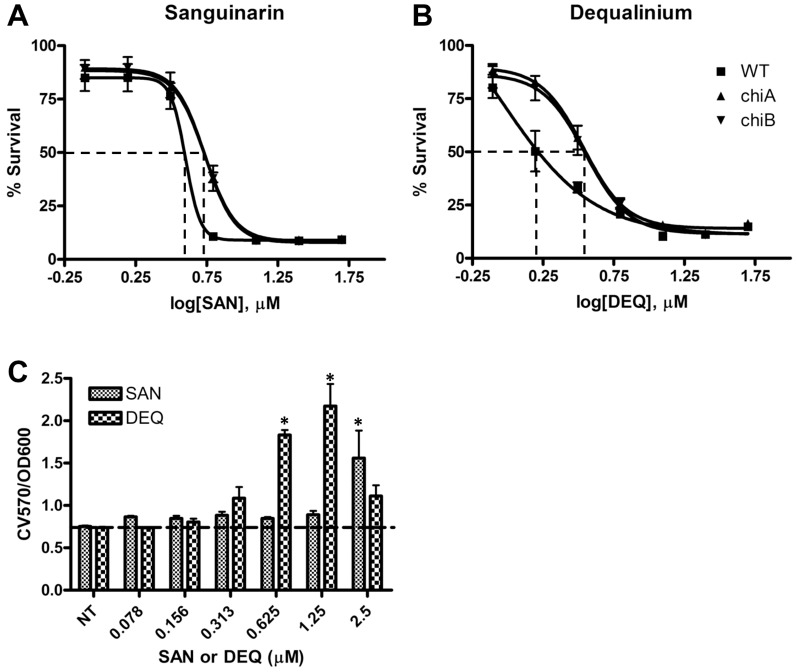
Effect of chitinase inhibitors SAN and DEQ on antibacterial and antibiofilm activity. (**A, B**) Susceptibility of *Fn* WT and *chi* mutants to SAN (**A**) and DEQ (**B**). Survival percentage of bacteria was calculated by OD_600_ measurements after 24 h incubation with various concentrations of SAN and DEQ in TSBC. The EC_50_s (μM) were determined by GraphPad software as indicated in the bottom table. (**C**) Effect of chitinase inhibitors SAN and DEQ on biofilm formation. Biofilm formation (CV570/OD600) was calculated by normalization with bacterial growth in each concentration of inhibitors. *P<0.05 compared to untreated (NT) control (n = 4).

To further examine whether chitinase activity itself is responsible for the anti-biofilm activity, we utilized potent family-18 chitinase inhibitors sanguinarine (SAN) and dequalinium (DEQ) which have inhibitory activity to chitinase and an antimicrobial activity to the bacteria [27]. The antimicrobial activity of SAN and DEQ was first tested against *Fn* WT, and the EC_50_s for SAN and DEQ were found to be 4.00 and 0.89 μM, respectively (Fig. 6). The *chi* mutants showed a slightly higher EC_50_ values (P<0.05) for SAN and DEQ, suggesting that the *chi* mutants with different surface charges responded differently to positive-charged SAN and DEQ in their drug susceptibility. To examine the effect of chitinase inhibitors on biofilm formation, we determined the anti-biofilm activity of the inhibitors on *Fn*. Fig. 6C demonstrates that biofilms of chitinase-positive WT, but not the *chi* mutants, were increased by treatment with chitinase inhibitors SAN and DEQ. This indicated that chitinase activity itself might be degrading the biofilms produced by *Fn* WT.

### Increased drug susceptibility of biofilms treated with exogenous chitinase

**Figure 7:**
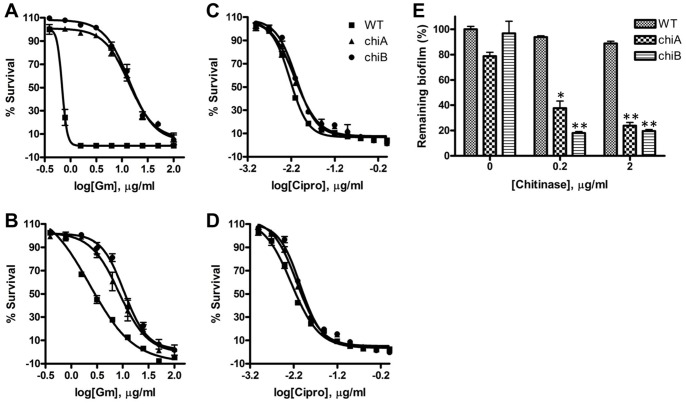
Chitinase alters drug susceptibility of *Fn* biofilms. (**A, C**) Effect of chitinase on drug susceptibility of biofilms pre-formed in the TC plates to (**A**) gentamicin (Gm) and (**C**) ciprofloxacin (Cipro). (**B, D**) Effect of chitinase on drug susceptibility of biofilms pre-formed in the amine plates to (**B**) gentamicin and (**D**) ciprofloxacin. (**E**) Susceptibility of chitinase-pretreated biofilms to gentamicin. Biofilms were formed on Amine plates in the presence of chitinase (0, 0.2 and 2 μg/ml) for 24 h then Gm (2 μg/ml) was added to the biofilms for 24 h. The remaining bacteria were calculated by the relative bacteria to no Gm-treated control in each concentration of chitinase. *P<0.05 compared to no Gm-treated control (n = 3).

Bacteria within biofilms are inherently resistant to antimicrobial agents. We therefore determined whether chitinase regulates the resistance of *Fn* biofilms to antimicrobial agents through its regulation of biofilm production. To examine drug susceptibility of the different strains, we cultured WT and *chiA* mutant in TC or amine plates. After 48 h of incubation, the wells were washed to remove planktonic bacteria and treated with gentamicin (10 μg/ml) for 24 h. The remaining live bacteria were quantified by resazurin reduction assays that detect cellular metabolic activity. WT *Fn* biofilms, which were thin in the TC plates (Fig. 2A), were highly sensitive (EC_50_  = 0.69 μg/ml), while the *chiA* mutant bacteria having thick biofilm formation on TC plates were highly resistant to gentamicin (EC_50_  = 13.77 μg/ml) (Fig. 7A). The EC_50_ of WT for gentamicin was 3-fold increased in the amine plates (EC_50_  = 2.2 μg/ml) compared with that in the TC plates (Fig. 7B) corresponding to the thicker biofilm (Fig. 2). The *chiA* biofilms grown on the TC and the amine plates did not exhibit significant differences in susceptibility to gentamicin. Of importance, difference of gentamicin sensitivity (4.7-fold in EC_50_) between WT and *chiA* mutants in the amine plates, despite showing no difference of biofilm formation between both strains, suggested that chitinase may sensitize the cells to gentamicin-mediated bacterial killing. On the other hand, WT and *chi*A biofilms grown on the TC and the amine plates did not exhibit significant differences in susceptibility to ciprofloxacin (Fig. 7C and D). Given that *chiA* biofilms are resistant to gentamicin, but not to ciprofloxacin, these results suggest that a highly charged aminoglycoside antibiotic gentamicin is delayed in biofilm penetration to kill the cells [28]. In addition, all three strains exhibited similar minimal inhibitory concentrations (1.56∼3.13 μg/ml) to gentamicin, suggesting that the *chi* mutants are more resistant, not just more tolerant, to cationic gentamicin.

To further elaborate the chitinase effect on drug susceptibility, WT and *chi* mutants were incubated overnight in the presence of chitinase (0, 0.2 and 2 μg/ml) and treated with gentamicin (2 μg/ml) for 24 h. After washing the detached or dead bacteria, the remaining bacteria in biofilms were quantified by resazurin reduction assays. Without chitinase treatment, bacteria were protected from gentamicin. However, exogenous chitinase treatment resulted in a drastic decrease of the remaining bacteria in the biofilm (Fig. 7E). This suggests that chitinase alters bacterial properties for drug susceptibility during biofilm formation.

### Chitinase modulates bacterial adhesion and invasion to A549 cells

**Figure 8:**
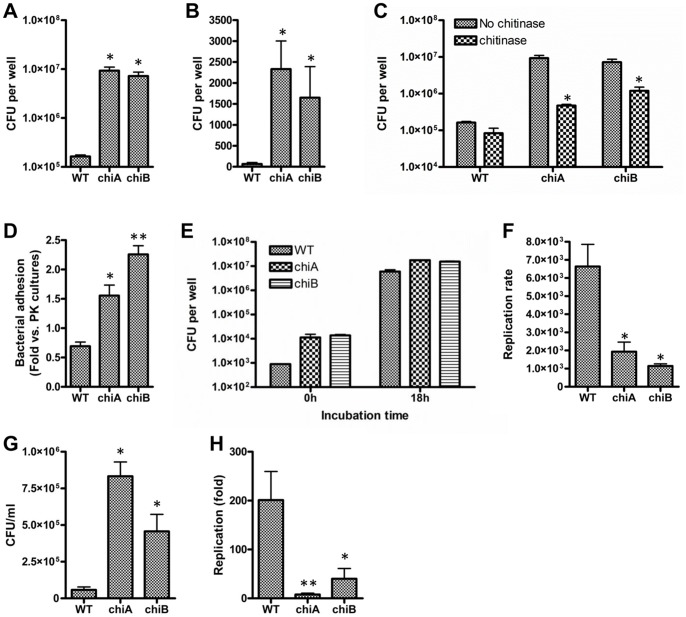
Abrogation of *Fn chi* genes enhances ability to adhere to, to invade to and to replicate in host cells. (**A**) Comparison of the adhesive properties of *Fn* WT and *chiA* mutants to A549 cells. *P<0.001 compared to WT (n = 6). (**B**) Bacterial invasion to A549 cells assayed by gentamicin protection method. *P<0.01 compared to WT (n = 6). (**C**) Effect of exogenous chitinase on bacterial adhesion. The same number of chitinase-treated bacteria as untreated bacteria were subjected to adhesion assays. *P<0.05 compared to untreated control of each strain (n = 3). (**D**) Bacterial adhesion assays using planktonic and biofilm cultures. Values are expressed as fold-increase adhesion relative to the planktonic counterparts. *P<0.05 and **P<0.01 compared to WT (n = 3). (**E**) Intracellular replication of the bacteria in host cells. A549 cells were infected with either WT or *chi* mutants at 100:1 MOI. Colony-forming units (CFUs) were determined after recovering intracellular bacteria from A549 cell lysates at 0 h or 18 h after gentamicin treatment to infected cells. (**F**) CFUs recovered from A549 cells lysed at 18 h post infection were compared with CFUs recovered at 0 h time point to calculate fold replication rate (change in CFU/hr). *P<0.05 compared to WT. (**G**) Bacterial invasion to J774A.1 cells assayed by gentamicin protection method. *P<0.01 compared to WT (n = 3). (**H**) Intracellular replication of the bacteria in J774A.1 cells. *P<0.05 and **P<0.01 compared to WT (n = 3).

Differential expression of extracellular matrix materials in biofilms alters adhesion and invasion of pathogens to host cells [29]. *In vitro* experiments including biophysical properties and drug susceptibility assays as described above suggested that chitinase changed the surface properties of *Francisella* in biofilms. We therefore investigated whether the matrix produced by the *chi* mutant bacteria could also promote bacterial adherence to and invasion of A549 human lung cell monolayers. As shown in Fig. 8A, the thick biofilm-forming *chi* mutants were able to adhere to the A549 cells ∼100-fold more than WT. Invasion of the bacteria as determined by gentamicin protection assay showed similar results as adhesion assays (Fig. 8B). To determine whether this activity was chitinase dependent, we performed the same experiment using chitinase-treated WT and *chi* mutant bacteria, which reduced biofilm formation. Treatment with chitinase before infection of the A549 cells resulted in a drastic decrease of adhesion ability of the *chi* mutants, while WT displayed only a slight decrease of adhesion in chitinase-treated bacteria compared to untreated control (Fig. 8C). Chitinase mutant bacteria showed 1.5–2.3 fold increase in adhesion in biofilm vs. planktonic form (Fig 8D). Within 24 h post infection, there were no drastic changes in A549 cell viability (Fig. S4). These results suggested that chitinase activity may modulate bacterial adhesion and invasion to the A549 cells through the change of surface matrix materials, which may be targets for chitinase.

In order to test the functional role of *Fn* chitinase, we determined intracellular replication of the mutants in A549 cells. The initial invasion of the *chi* mutants was significantly higher than that of WT *Fn*; however, there was no significant difference in intracellular bacteria at 18 h post infection (Fig. 8E). Calculation of replication rates from Fig. 8E implied that the *chi* mutants may have a severe defect (3.7∼5.7-fold of decrease) in replication rates compared with WT 18 h post infection (Fig. 8F). Invasion activity and intracellular replication rates of the mutants in J774A.1 cells had a similar pattern to the A549 cells (Fig. 8G and 8H), supporting the conclusion obtained from A549 cells. Overall, these data suggested that *Fn* chitinase might be involved in some pathogenic function of the pathogen, although its overall contribution to virulence is not clearly observed in murine model [8], [30], [31].

## Discussion

In previous studies, we demonstrated that *Fn*, a model organism of highly virulent *F. tularensis*, forms a biofilm *in vitro,* mediated by an orphan response regulator [7]. We also reported that *F. philomiragia*, which causes francisellosis of farmed and wild fish, can form a biofilm in a co-culture with *Acanthamoeba castellanii*, an aquatic amoeba [9], and suggested these biofilms may be ‘lures’ for environmental amoebae and other protists. Margolis *et al.* [8] showed that *Fn* forms biofilms during the colonization of chitin surfaces (i.e. crab shells) by using chitin as a sole carbon source. They demonstrated that mutants lacking *chiA* or *chiB* were attenuated for chitin colonization and biofilm formation in the absence of exogenous sugar. *Fn* secretes proteins including chitinases (ChiA and ChiB), a chitin binding protein (CbpA), a protease (PepO), and a beta-glucosidase (BglX) [32]. In the present study, we show that chitinase modulates attachment and biofilm formation on abiotic material and host cell surfaces.

Bacterial surface characteristics are important in bacterial attachment to substrates [17], [33]. To understand which surface properties might play an important role in the initial attachment of *Fn* to substrates, we compared fundamental surface properties (i.e., hydrophobicity and surface charges) of WT and *chi* mutants. Results indicated that WT bacteria are more hydrophobic and less charged than *chiA* or *chiB* mutants. However, autoaggregation rate are higher in *chi* mutants compared to WT bacteria, suggesting that a more charged bacterial surface may contribute to cell-to-cell interaction for aggregation. These properties might partly account for the increased resistance of *chiA* mutants to cationic antimicrobial gentamicin. Surface charges significantly affect initial attachment and biofilm formation. Different surface charged microplates were used to demonstrate the relationship between WT and *chi* mutant initial attachment and subsequent biofilm formation. There was no difference between WT and mutants in the positively-charged (amine plate), uncharged hydrophobic (polystyrene plate), and net zero-charged surface (Primaria plate). In contrast, *chi* mutants exhibited high biofilm formation on the negatively-charged tissue-culture plates compared to WT bacteria. Exogenous addition of chitinase protein could explain, in part, the effect of chitinase gene on biofilm formation. In addition, since Margolis *et al.* [8] showed that addition of the *chiA* and *chiB* genes to deletion mutant strains complemented the chitin colonization defects, these results suggest that chitinase modulates surface charge of bacteria, resulting in high attachment and biofilm formation to the negatively-charged surface. This charge-dependent biofilm formation may contribute to defining the natural environments for *Francisella* biofilm formation.

Furthermore, such surface properties might be linked to bacterial adhesion and invasion to host cells [34], [35]. *Chi* mutants showed a dramatic increase of adhesion and invasion to human lung epithelial A549 cells compared to WT. Our data showed that replication rate of *chi* mutants, on the other hand, was decreased in A549 cells. One may speculate that positively-charged *chi* mutant bacteria are able to more efficiently bind negatively-charged A549 cell membranes [36], resulting in an increased invasion activity. However, Mellio *et al.* [34] have reported that *F. tularensis* surface protein FsaP was able to bind A549 cells. Although we did not examine whether chitinase mutation induced FsaP expression to investigate a link between them, changes of surface charges by chitinase may contribute to adhesion and invasion changes. This explanation is supported by our finding that addition of exogenous chitinase to WT bacteria decreased bacterial adhesion to A549 cells.

Our data also showed that chitinase is involved in the detachment of pre-formed biofilms by its enzymatic activity. This implies that *Francisella* biofilms include a substrate for chitinase in extracellular matrix. The chitinase substrate chitin is the second most abundant natural polysaccharide consisting of β (1→ 4)-linked *N*-acetyl-D-glucosamine (GlcNAc) units in a linear form. There are no reports of chitin production in *Francisella* species; however, chitinase is required for providing carbon source under nutrient-limiting conditions [37]. Nevertheless, our detachment studies of pre-formed *Francisella* biofilm with chitinase imply a possibility for existence of a yet-unrecognized chitinase substrate in biofilms. The content and composition of the potential substrates could be different between WT and *chi* mutants based on the differential susceptibility of the bacteria to exogenous chitinase (Fig. 5A and 5B). This speculation is also supported by resistance of WT biofilms to gentamicin compared to that of *chi* mutants (Fig. 7).

Recently, Margolis *et al.* [8] showed that ChiA and ChiB are important for *Fn* biofilm formation on biotic chitinous crab shell surfaces. This finding was also confirmed on abiotic glass surfaces [38]. In another study, however, *chiA* and *chiB* mutants showed no defects in the ability to colonize ticks [39], which have chitin in their exoskeleton. Our study using abiotic, different-charged polystyrene microplates showed that, unlike on glass or crab shells, there was no difference between WT and *chi* mutants; however, negatively-charged tissue-culture plate and human lung epithelial cells showed increased biofilm formation in *chi* mutants. This suggests that regulation of biofilm formation by *Fn* chitinase is sensitive to environmental conditions, i.e. charges of substrata and bacterial surfaces.

In summary, we have shown that chitinase plays a pivotal role in biofilm formation by *Fn*. Substratum surface charges affect *Fn* biofilm formation. Changes of biophysical properties (hydrophobicity, surface charge, and autoaggregation) by *chi* mutation increased *Fn* biofilm formation. Preformed *Fn* biofilms were degraded by treatment with protease K, chitinase and DNase. Chitinase-treated preformed biofilms became susceptible to gentamicin killing. In addition, mutation of *chi* genes enhanced bacterial adhesion and invasion to A549 and J774A.1 cells; however, intracellular replication rate was decreased in *chi* mutants. We propose that regulation of EPS may be involved in chitinase-mediated biofilm formation and bacterial invasion of the host.

## Materials and Methods

### Bacterial strains and growth conditions

*F. tularensis* ssp. *novicida* type strain U112 (*Fn*) wild-type (WT), *Fn chiA* (NR-5007) and *chiB* (NR-6005) transposon mutants, *Pseudomonas aeruginosa* (Schroeter) Migula R. Hugh 813 and *Staphylococcus aureus* ssp. *aureus* Rosenbach 502A were obtained from American Type Culture Collection (Manassas, VA). *Francisella* strains were cultured at 37°C in tryptic soy broth containing 0.1% cysteine (TSBC). Kanamycin (20 μg/ml) was used to select for *Fn* mutants. *P. aeruginosa* and *S. aureus* were cultured at 37°C in nutrient broth. To validate the *Tn* mutation of the *chi* gene, we performed PCR with genomic DNA and qRT-PCR with total RNAs isolated from each strain. The results confirmed the mutation of *chiA* and *chiB* gene by a transposon insertion using primers of the outside of flanking region (Fig. S5A and S5B). Chitinase activity in culture supernatants was also decreased by a transposon insertion, especially in chitobiosidase activity (Fig. S5C). Of note, there was no significant β-N-acetyl-glucosaminidase activity in *Fn* culture supernatants and the endochitinase activity was increased in both *chi* mutants. There was no growth defect in the *chi* mutants compared with WT (Fig. S5D).

### Crystal violet assays for biofilms

Biofilm was measured as previously described [7] with the following modifications. Bacteria (1×10^6^ per well) in 100 μl of TSBC were incubated without and with antibiotics for 24–48 h at 37°C in different types of 96-well microplate: negatively-charged carboxyl group-containing, tissue-culture (TC)-treated polystyrene (PS); nontreated, hydrophobic PS; positively-charged, amine group-containing PS; and both negatively and positively-charged, carboxyl and amine group-containing Primaria PS (BD Biosciences). Optical density of the cultures (OD_600_) was determined prior to staining as a measure of bacterial growth. Biofilm production was measured using the crystal violet (CV) stain technique with absorbance measurements at 570 nm (CV_570_) [6]. For bacterial attachment assays, overnight culture (OD_600_  = 1.0) of bacteria in 100 μl of TSBC was incubated in different types of plates for 1 h at 37°C. Attached bacteria were measured using the CV stain technique [7], [8].

### Confocal Scanning Laser Microscopy (CSLM)

Bacteria were grown on the Lab-Tek II chamber slide (Thermo Scientific) for 24 h and biofilms attached to the glass were fixed with methanol followed by DAPI staining (920 ng/ml). Biofilm structure was observed using a Nikon TE2000-U confocal laser scanning microscope equipped with an argon ion laser. Sections through the XY, YZ and XZ planes were obtained using the Nikon EZ-C1 Confocal Software program. Each strain was examined on at least three separate occasions. Quantitative analysis of the CLSM z-stacks was performed using established protocols for the image analysis tool COMSTAT2 [40], [41]. The biofilm parameters, biomass (biovolume), mean thickness, and roughness coefficient (an indicator of biofilm heterogeneity) were assessed using a minimum of 3 different images per plate from 2 independent experiments for each strain.

### Hydrophobic interaction chromatography (HIC)

The cell surface hydrophobicity of WT and *chi* mutants was determined using a Pasteur pipette with 1 ml of phenyl sepharose fast flow resin (GE Healthcare), washed with 50 mM phosphate buffer (pH 7.2) containing 150 mM NaCl. Bacteria (cultured overnight) were diluted 10-fold in TSBC, incubated for 4 h for mid-log phase culture. Bacteria were washed and resuspended with 50 mM phosphate buffer (pH 7.2), 150 mM NaCl. Bacteria (0.3 ml) were loaded onto column, washed with 0.9 ml of the same buffer. OD_600_ was measured (PowerWave X microplate reader, BioTek Instruments), and the percentage of bacteria retained in the hydrophobic column was calculated from the absorbance of a ¼ dilution of the original bacterial suspension as follows. **Equation 1**: % adsorption  =  [(A_0_-A_1_)/A_0_] x 100, where A_0_  =  OD of ¼ diluted bacterial suspension, and A_1_  =  OD of the eluted bacterial suspension.

### Microbial adhesion to hydrocarbon test (MATH)

Relative cell-surface hydrophobicity was measured by microorganism adhesion to hydrocarbon hexadecane (Sigma-Aldrich). Mid-log phase culture (50 mM phosphate buffer, pH 7.2) as prepared above was added to equal volume of hexadecane, vortexed for 30 s, and incubated for 20 min at room temperature. OD_600_ of the aqueous phase was measured and percentage adsorption was determined using **Eq. 1**.

### Autoaggregation

Autoaggregation was measured according to published method [42]. Briefly, cells harvested at stationary phase were washed twice with PBS (pH 7.4), resuspended in PBS (5 ml) to OD_600_ ∼1.0. The tubes were stored at room temperature and OD_600_ of the upper 0.5 ml culture was measured at 0, 24 and 48 h. Percentage of autoaggregation was calculated as described above (**Eq. 1**).

### qNano analysis of bacteria

Relative surface charge and size distribution analysis of WT, and mutants *chiA* and *chiB* was performed using a qNano (Izon Science). The qNano utilizes Tunable Resistive Pulse Sensing technology to allow for a high-throughput, particle-by-particle, analysis of particle size, surface charge, and electrophoretic mobility [20], [43]. All qNano experiments were performed using the manufacturer's established protocols [19]–[21], [43]. Briefly, overnight cultures of WT, *chiA*, and *chiB* were pelleted (five minutes at 4,500×g) and washed three times with sterile PBS. For each measurement, 40 μl of the washed bacterial suspension was added to the top fluid cell and a minimum of 1,000 blockade events were recorded. Measurements were taken at 48.49 mm of applied stretch with an applied voltage of 0.10 V. An applied pressure of 5 cm H_2_O was applied to the top fluid cell using the Izon Science variable pressure module. The size distribution and relative surface charge analysis was performed using IZON proprietary software V2.2.

### EPS determination

For EPS extraction, 30 ml of culture was pelleted and resuspended in 1/5 volume of TNE (10 mM Tris-HCl, pH 7.5, 5 mM EDTA, 100 mM NaCl) containing 0.1% SDS (final). The samples were stirred for 5 min at room temperature, passed 5 times through 18-G needle, and then centrifuged at 17,000×g for 15 min. The pellets were washed 5 times with 10 mM Tris-HCl (pH 7.5) and resuspended in 3 ml 10 mM Tris-HCl (pH 7.5). Carbohydrate content was determined by phenol-sulfuric acid method with glucose standard [44]. Briefly, 50 μl of sample or standard was added to 150 μl conc. H_2_SO_4_ and 30 μl of 5% phenol in water. The samples were heated at 90°C for 5 min in a hot plate and cooled down to RT. The absorbance values at 490 nm were used to determine released carbohydrate (in mg per g cells or ml of culture supernatants).

### Lectin binding assays

Bacteria were cultured overnight in TSBC medium in a 96 well or a 6-well TC plate. The wells were washed 3 times with PBS and incubated with FITC-labeled ConA (Invitrogen; a final concentration of 50 μg/ml in PBS) for 30 min. After washing 5 times with PBS, fluorescence of the well in a 96 well plate was measured by Tecan Safire II microplate reader (Tecan) with excitation of 488 nm and emission of 532 nm (bandwidth  = 10 nm) and images of the well in a 6-well plate were taken by EVOS FL Cell Imaging System (Life Technologies) using green channel, respectively.

### Enzymatic detachment of biofilms

Proteinase K from *Trichoderma album,* chitinase from *Streptomyces griseus,* and DNase I (Sigma-Aldrich) were used. Biofilm disruption by proteinase K and chitinase was assayed in 50 mM sodium phosphate buffer (pH 7.2); that of DNase I was in 10 mM Tris-HCl (pH 7.6), 2.5 mM MgCl_2_ and 0.5 mM CaCl_2_. Pre-formed biofilms (2 days in the TC plates) were incubated with 50 μg/ml of each enzyme and control received an equal volume of buffer without enzyme. The plates were incubated for 2 h at 37°C, biofilms stained by CV as described above.

### Antimicrobial susceptibility

Antimicrobial susceptibility of biofilm was performed by the resazurin reduction assay. Bacterial biofilm was formed (1×10^6^ CFU per well in the TC or amine plate, incubated for 48 h at 37°C). The wells were gently washed twice with PBS. 100 μl TSBC containing serially diluted from 10 μg/ml gentamicin was added, incubated 24 h. Wells were washed twice with PBS and cation adjusted Mueller Hinton broth was added containing 50 μg/ml of resazurin (R&D Systems). Plates were incubated for 2 h at 37°C and the absorbance was measured at 570 nm with a reference at 600 nm. Percent survival was calculated by **Eq. 2**. For determining the effect of chitinase on gentamicin susceptibility of biofilm-encased organisms, bacteria were cultured overnight in the amine plates in the presence of 0.2 and 2 μg/ml of chitinase in TSBC. The wells were washed 3 times with PBS and added gentamicin (0 and 2 μg/ml) in TSBC and incubated overnight. The remaining bacteria were assayed by the resazurin reduction method. **Equation 2**: % survival  =  (OD_570_ of gentamicin-treated sample)/(OD_570_ of none-treated control sample) ×100.

### Adhesion and invasion assays

Adhesion and invasion of *Fn* WT and *chi* mutants to A549 cells and J774A.1 cells were analyzed as described [35]. Briefly, 1×10^5^ A549 cells (ATCC, Cat. No. CCL-185) in Ham's F-12K (10% fetal bovine serum) or J774A.1 cells (ATCC, TIB-67) in RPMI1640 (10% fetal bovine serum) were incubated overnight in a 24-well tissue-culture plate (37°C with 5% CO_2_). Bacteria (multiplicity of infection of 100∶1) were added to the monolayers in a final volume of 500 μl. Plates were centrifuged at 800×g for 5 min to synchronize the infection followed by a 2 h incubation at 37°C with 5% CO_2_. Cell monolayers were washed five times with PBS to remove non-adherent bacteria. Cells were disrupted by the addition of 0.1% deoxycholic acid solution in PBS. The total number of cell associated bacteria was enumerated by serial plating on TSBC agar plates. To quantify the number of intracellular bacteria, cell monolayers were incubated with bacteria for 2 h followed by the addition of gentamicin (20 μg/ml) for 1 h to kill extracellular bacteria. The monolayers were washed a total of six times in PBS, lysed by 0.1% deoxycholic acid in PBS and plated on TSBC agar. To examine the effect of chitinase, overnight cultured bacteria were diluted 20-fold in TSBC containing 20 μg/ml chitinase for 3 h. After enumerating bacterial number by measuring OD, bacteria were subjected to adhesion assays as described. All assays were performed in triplicate and repeated at least twice in independent experiments.

### Statistics

All experiments were performed in biological triplicate and repeated at least twice in independent experiments. All data were expressed as arithmetic means ± standard deviations. Comparisons between groups were carried out using the unpaired Student's t-test. P-values were determined by the unpaired Student t-test using Excel and GraphPad Prism software. Statistical significance was set at P<0.05.

## Supporting Information

Figure S1 **(A)** Crystal violet staining of biofilms grown in a tissue culture plate (carboxyl, BD PureCoat) for 24 and 48 h. **(B)** One hour attachment to different microplates. Bacteria were allowed to attach for 1 h following published procedure [8]. Initial attachment of *chi* mutants was compared with that of WT to calculate fold increase in each plate. *P<0.01 compared to WT (n = 6). **(C)** Representative light microscopic images of *Fn* WT, *chiA* and *chiB* mutant attachment to the TC plates after CV staining. **(D)** Representative confocal laser scanning microscopic (CLSM) images of *Fn* WT, *chiA* and *chiB* mutant attachment to the borosilicate glass chamber after DAPI staining (pseudocolored red). For each panel, the large, center image is a single section in the xy plane, the lower image is the xz plane, and the right image is the yz plane. White lines indicate the particular locations of the xz, yz, and xy planes depicted.
(TIF)

Figure S2 **Chitinase activity from** ***F. novicida*** **(recombinant ChiA),** ***S. griseus, and Trichoderma viridae*** **.** The assay was based on the release of 4-methylumbelliferone (4MU) by enzymatic hydrolysis from chitinase substrates, 4-methylumbelliferyl N,N′-diacetyl-β-D-chitobioside for chitobiosidase activity, 4-methylumbelliferyl N-acetyl-β-D-glucosaminide for β-N-acetylglucosaminidase activity, and 4-methylumbelliferyl β-D-N,N′,N″-triacetylchitotriose for for endochitinase activity.
(TIF)

Figure S3 Effect of chitinase on biofilm formation of *S. aureus* and *P. aeruginosa*, *S. aureus* and *P. aeruginosa* were cultured in the TC plates for 2 days in the presence of different concentrations of chitinase. Biofilm production was measured using the CV stain technique with absorbance measurements at 570 nm (CV_570_). Relative biofilm formation of each condition was calculated by dividing CV_570_ of untreated control.
(TIF)

Figure S4 **No cytotoxic effect of** ***F. novicida*** **infection on A549 cells for 24 h.** Lactate dehydrogenase (LDH) released into the supernatant was measured using a commercially available kit (Promega CytoTox 96 Non-Radioactive Cytotoxicity Assay). Cytotoxicity was expressed relative to LDH release from whole cell lysates in controls (n = 6).
(TIF)

Figure S5 **Confirmation of** ***Fn chi*** **mutants.** (**A**) Colony PCR of mutants. Single colony of each strain was suspended in water (100 μl) and 2 μl of cell suspension was subjected to a typical PCR reaction (20 μl) with forward primer (5′-ACAGCACCAATGTTTGAGCA-3′) and reverse primer (5′-CAATACGACTTCTCGCACCA-3′) for *chiA* and forward primer (5′- TCTGTAAATCTAACTGGTGATA-3′) and reverse primer (5′-ACTTATACTAATTGTGTAGTT A-3′) for *chiB* mutants. (**B**) qRT-PCR of total RNAs isolated from WT, *chiA* and *chiB* mutants using SYBR green dye and the same primers. (**C**) Chitinase assays of culture supernatants prepared from overnight cultures using fluorimetric chitinase assay kit (Sigma). *P<0.01 and **P<0.001 compared to WT (n = 3). (**D**) Growth kinetics of WT and *chi* mutants in TSBC media using a PowerWave X microplate reader (BioTek Instruments) with kinetic mode.
(TIF)
