# Supplementary figures and images for: Chitinases Are Negative Regulators of Francisella novicida Biofilms
Source: PLoS One. 2014 Mar 24;9(3):e93119. doi: 10.1371/journal.pone.0093119 (PMC3963990; doi:10.1371/journal.pone.0093119)

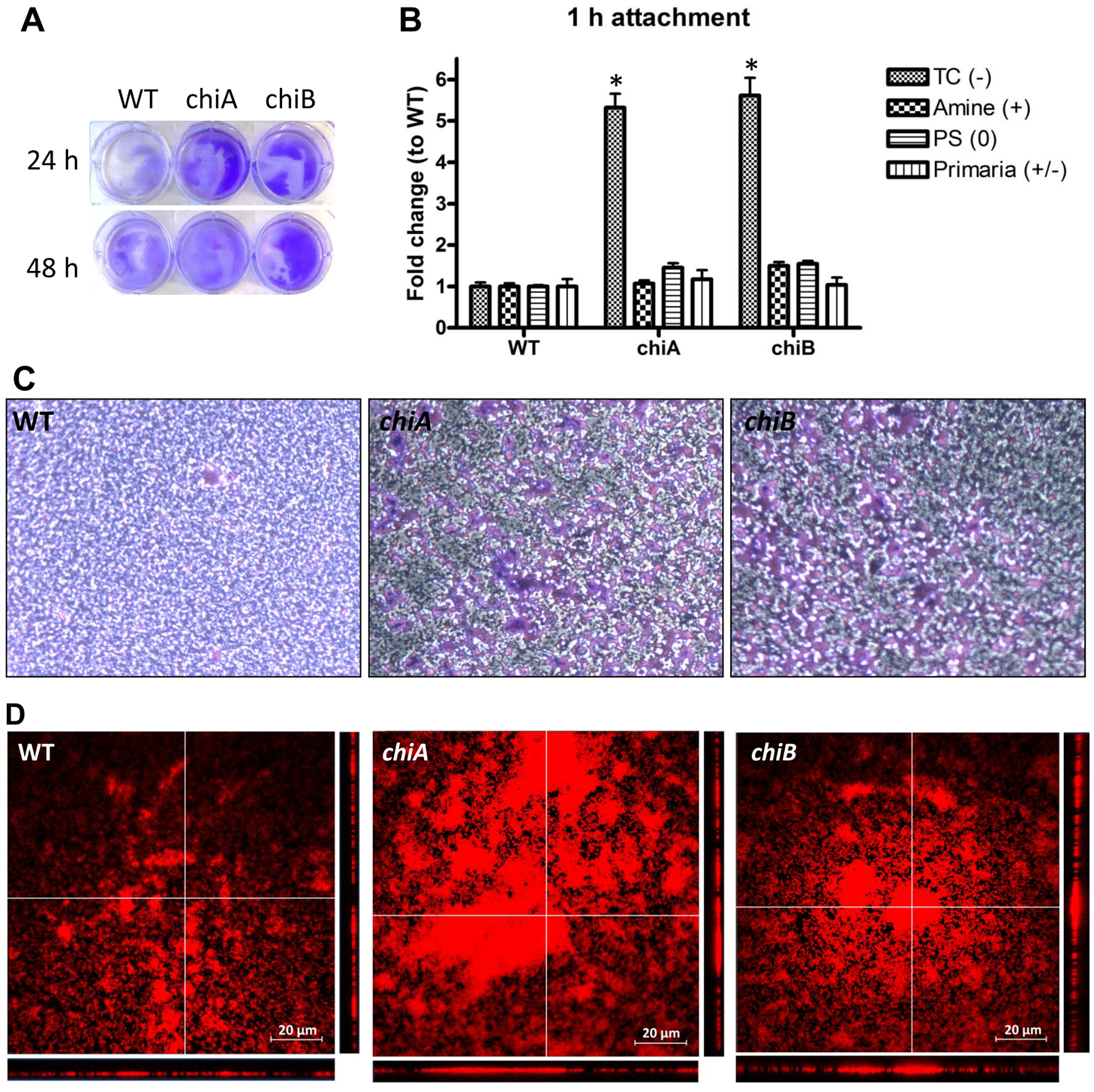

Supplement: Figure S1 — (A) Crystal violet staining of biofilms grown in a tissue culture plate (carboxyl, BD PureCoat) for 24 and 48 h. (B) One hour attachment to different microplates. Bacteria were allowed to attach for 1 h following published procedure [8]. Initial attachment of chi mutants was compared with that of WT to calculate fold increase in each plate. *P<0.01 compared to WT (n = 6). (C) Representative light microscopic images of Fn WT, chiA and chiB mutant attachment to the TC plates after CV staining. (D) Representative confocal laser scanning microscopic (CLSM) images of Fn WT, chiA and chiB mutant attachment to the borosilicate glass chamber after DAPI staining (pseudocolored red). For each panel, the large, center image is a single section in the xy plane, the lower image is the xz plane, and the right image is the yz plane. White lines indicate the particular locations of the xz, yz, and xy planes depicted. (TIF) [file pone.0093119.s001.tif]

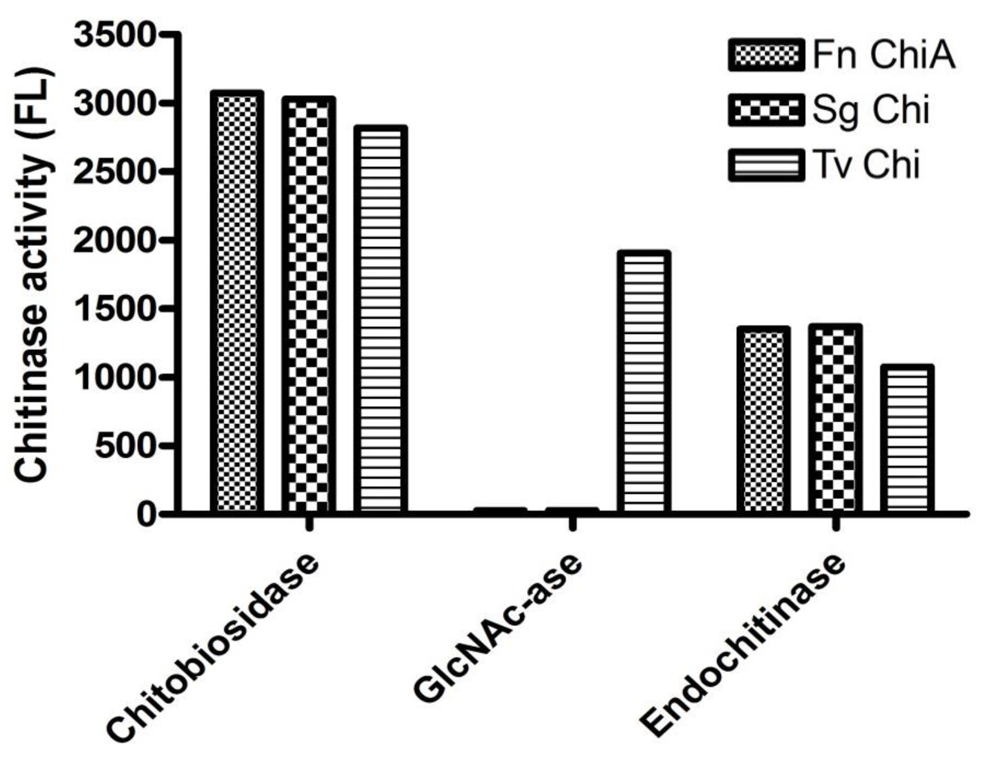

Supplement: Figure S2 — Chitinase activity from F. novicida (recombinant ChiA), S. griseus, and Trichoderma viridae . The assay was based on the release of 4-methylumbelliferone (4MU) by enzymatic hydrolysis from chitinase substrates, 4-methylumbelliferyl N,N′-diacetyl-β-D-chitobioside for chitobiosidase activity, 4-methylumbelliferyl N-acetyl-β-D-glucosaminide for β-N-acetylglucosaminidase activity, and 4-methylumbelliferyl β-D-N,N′,N″-triacetylchitotriose for for endochitinase activity. (TIF) [file pone.0093119.s002.tif]

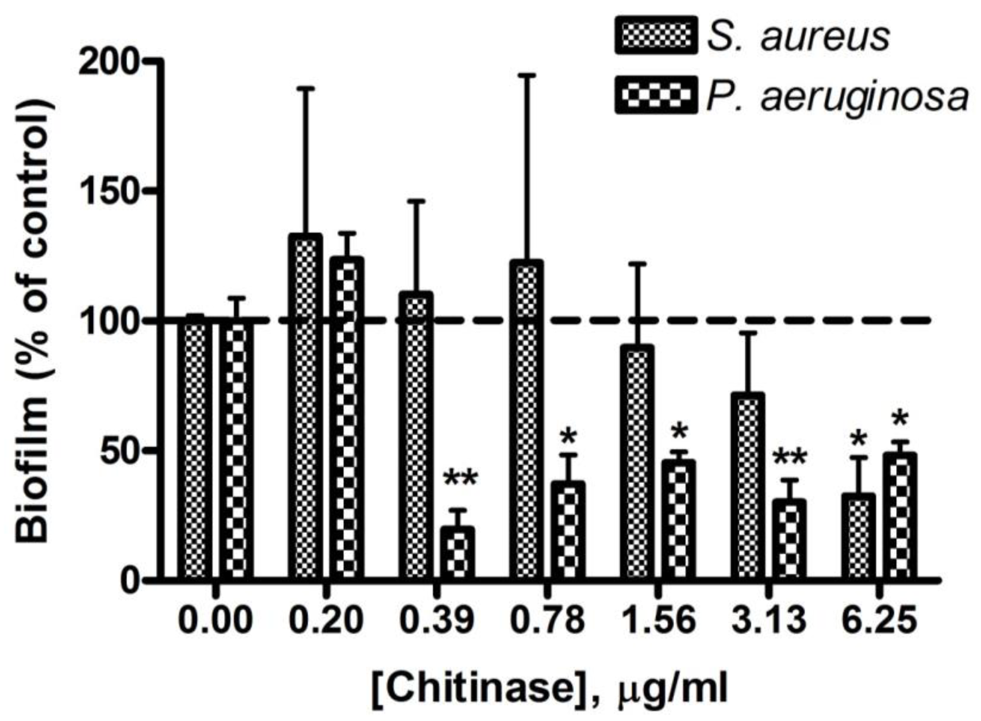

Supplement: Figure S3 — Effect of chitinase on biofilm formation of S. aureus and P. aeruginosa, S. aureus and P. aeruginosa were cultured in the TC plates for 2 days in the presence of different concentrations of chitinase. Biofilm production was measured using the CV stain technique with absorbance measurements at 570 nm (CV570). Relative biofilm formation of each condition was calculated by dividing CV570 of untreated control. (TIF) [file pone.0093119.s003.tif]

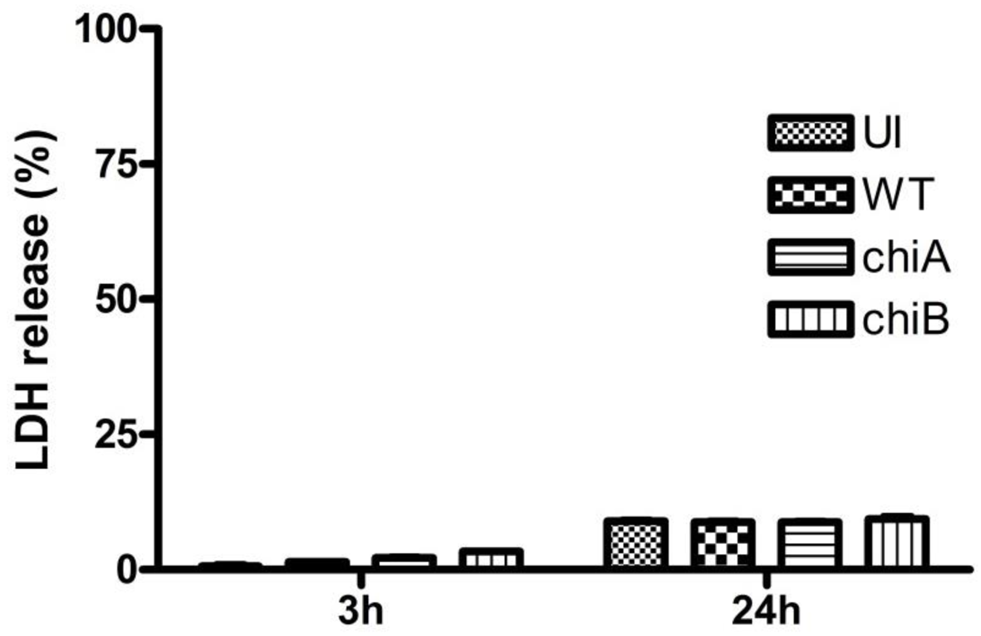

Supplement: Figure S4 — No cytotoxic effect of F. novicida infection on A549 cells for 24 h. Lactate dehydrogenase (LDH) released into the supernatant was measured using a commercially available kit (Promega CytoTox 96 Non-Radioactive Cytotoxicity Assay). Cytotoxicity was expressed relative to LDH release from whole cell lysates in controls (n = 6). (TIF) [file pone.0093119.s004.tif]

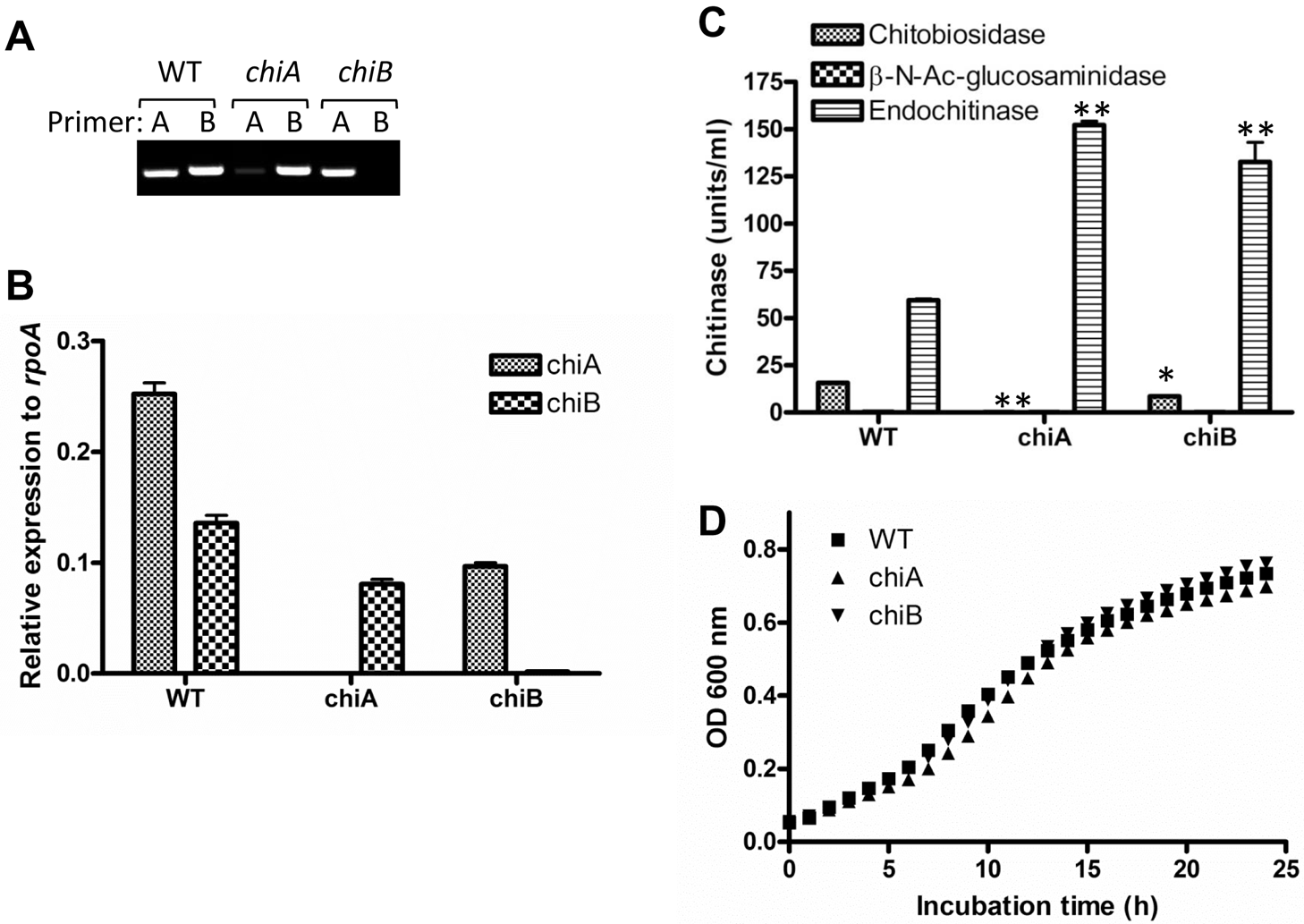

Supplement: Figure S5 — Confirmation of Fn chi mutants. (A) Colony PCR of mutants. Single colony of each strain was suspended in water (100 μl) and 2 μl of cell suspension was subjected to a typical PCR reaction (20 μl) with forward primer (5′-ACAGCACCAATGTTTGAGCA-3′) and reverse primer (5′-CAATACGACTTCTCGCACCA-3′) for chiA and forward primer (5′- TCTGTAAATCTAACTGGTGATA-3′) and reverse primer (5′-ACTTATACTAATTGTGTAGTT A-3′) for chiB mutants. (B) qRT-PCR of total RNAs isolated from WT, chiA and chiB mutants using SYBR green dye and the same primers. (C) Chitinase assays of culture supernatants prepared from overnight cultures using fluorimetric chitinase assay kit (Sigma). *P<0.01 and **P<0.001 compared to WT (n = 3). (D) Growth kinetics of WT and chi mutants in TSBC media using a PowerWave X microplate reader (BioTek Instruments) with kinetic mode. (TIF) [file pone.0093119.s005.tif]
